# Nanoparticles in the Treatment of Infections Caused by Multidrug-Resistant Organisms

**DOI:** 10.3389/fphar.2019.01153

**Published:** 2019-10-04

**Authors:** Nan-Yao Lee, Wen-Chien Ko, Po-Ren Hsueh

**Affiliations:** ^1^Department of Internal Medicine and Center for Infection Control, National Cheng Kung University Hospital and Medical College, Tainan, Taiwan; ^2^Department of Medicine, College of Medicine, National Cheng Kung University Hospital, Tainan, Taiwan; ^3^Department of Laboratory Medicine, National Taiwan University Hospital, National Taiwan University College of Medicine, Taipei, Taiwan; ^4^Department of Internal Medicine, National Taiwan University Hospital, National Taiwan University College of Medicine, Taipei, Taiwan

**Keywords:** nanoparticle, antimicrobial resistance, pharmacokinetics, pharmacodynamics, toxicity

## Abstract

Nanotechnology using nanoscale materials is increasingly being utilized for clinical applications, especially as a new paradigm for infectious diseases. Infections caused by multidrug-resistant organisms (MDROs) are emerging as causes of morbidity and mortality worldwide. Antibiotic options for infections caused by MDROs are often limited. These clinical challenges highlight the critical demand for alternative and effective antimicrobial strategies. Nanoparticles (NPs) can penetrate the cell membrane of pathogenic microorganisms and interfere with important molecular pathways, formulating unique antimicrobial mechanisms. In combination with optimal antibiotics, NPs have demonstrated synergy and may aid in limiting the global crisis of emerging bacterial resistance. In this review, we summarized current research on the broad classification of the NPs that have shown *in vitro* antimicrobial activity against MDROs, including the ESKAPE pathogens (*Enterococcus faecium*, *Staphylococcus aureus*, *Klebsiella pneumoniae*, *Acinetobacter baumannii*, *Pseudomonas aeruginosa*, and *Enterobacter* species). The pharmacokinetics and pharmacodynamic characteristics of NPs and bacteria-resistant mechanisms to NPs were also discussed.

## Introduction

Multidrug-resistant organisms (MDROs) are becoming a growing public health crisis and make many healthcare-associated infections difficult to treat with current antibiotics (Boucher et al., 2009; Peleg and Hooper, 2010). Globally, infections caused by MDROs are emerging causes of morbidity and mortality (Ismail et al., 2018; Kuo et al., 2018; Ting et al., 2018; Tsao et al., 2018). The development of new antibiotics requires tremendous economic and labor investment and is time-consuming (Huh and Kwon, 2011). For these MDRO infections, high doses of antibiotics will be administered and may generate intolerable toxic and adverse effects, which will prompt the development of alternative strategies.

The application of nanoparticles (NPs) provides a potential strategy to manage infections caused by MDROs (Singh et al., 2014; Natan and Banin, 2017; Baptista et al., 2018; Muzammil et al., 2018). In this respect, NPs have shown therapeutic promise owing to their unique physical and chemical attributes (Pelgrift and Friedman, 2013; Beyth et al., 2015; Hemeg, 2017). NPs exhibiting antibacterial activities can target multiple biomolecules and have the potential to reduce or eliminate the evolution of MDROs (Slavin et al., 2017). However, the translation of NPs to clinical use requires not only appropriate methods for the synthesis of NPs but also a thorough understanding of the physicochemical particularities, *in vitro* and *in vivo* effects, biodistribution, pharmacokinetics, and pharmacodynamics of NPs (Burdusel et al., 2018).

In this review, we will present a broad classification of the NPs that show *in vitro* antimicrobial activity against MDROs, and the synergistic effects of NPs with current available antibiotics, pharmacokinetic and pharmacodynamic characteristics, and resistant mechanisms will also be discussed.

## Applications of NPs as Antimicrobial Agents

**Figure 1:**
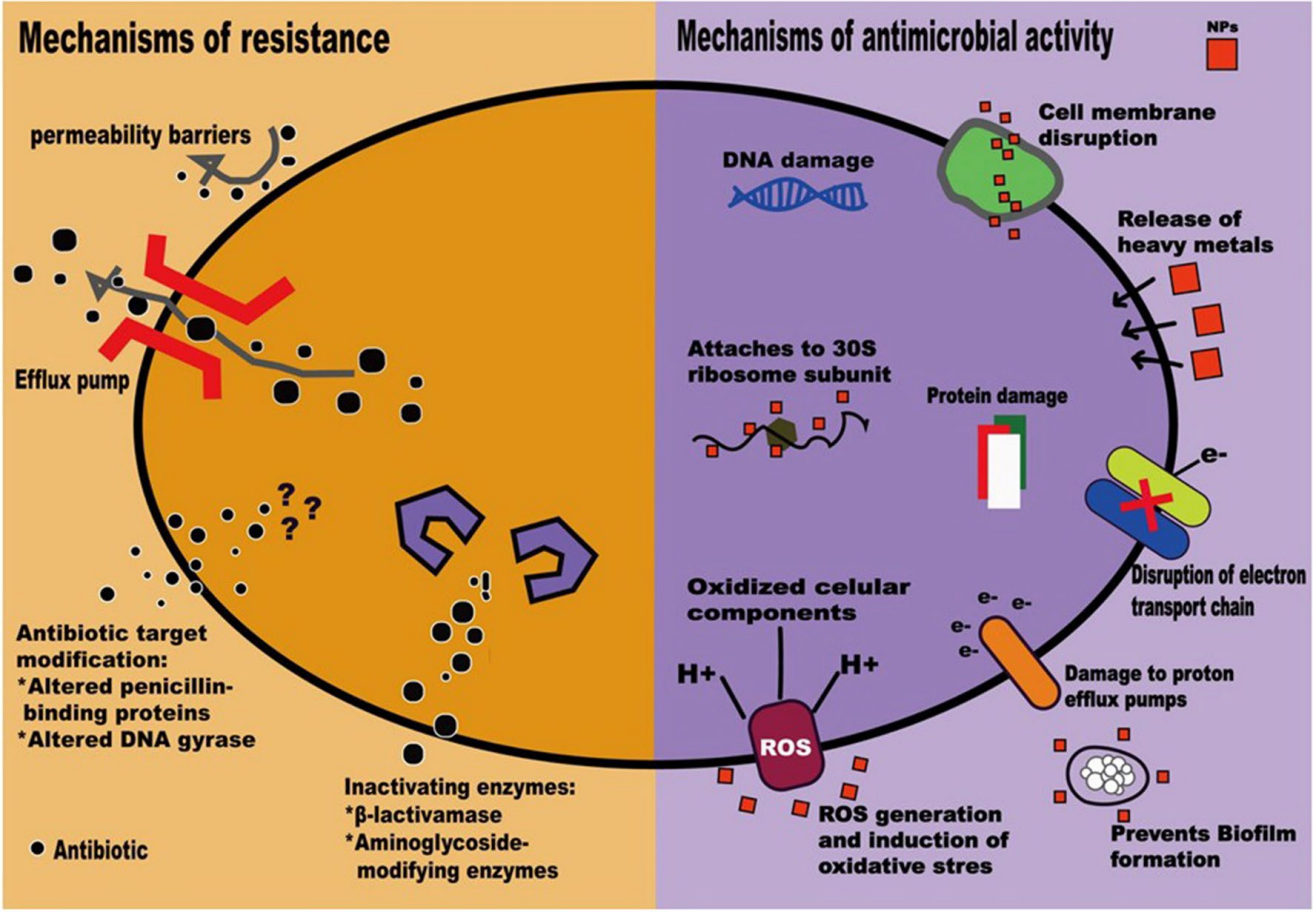
Mechanisms of antimicrobial resistance (Mulvey and Simor, 2009) and actions of nanoparticles (Baptista et al., 2018).

NPs possess antimicrobial activity that can overcome common resistant mechanisms, including enzyme inactivation, decreased cell permeability, modification of target sites/enzymes, and increased efflux through overexpression of efflux pumps, to escape from the antibacterial activity of antimicrobial agents (Mulvey and Simor, 2009; Baptista et al., 2018) (**Figure 1**). Moreover, NPs conjugated with antibiotics show synergistic effects against bacteria, prohibit biofilm formation, and have been utilized to combat MDROs (Pelgrift and Friedman, 2013; Baptista et al., 2018).

Several characteristics of NPs make them alternatives to traditional antibiotics. First, the large surface-area-to-volume ratio of NPs increases the contact area with target organisms. NPs can act as nanoscale molecules interacting with bacterial cells, regulating cell membrane penetration, and interfering with molecular pathways (Rai et al., 2012; Dakal et al., 2016; Duran et al., 2016; Hemeg, 2017). Second, NPs may enhance the inhibitory effects of antibiotics. Saha et al. (2007) demonstrated that gold NPs conjugated with ampicillin, streptomycin, or kanamycin could lower the minimum inhibitory concentrations (MICs) of the antibiotic counterparts against both gram-negative and gram-positive bacteria. Likewise, Gupta et al. (2017) demonstrated a synergistic effect of functionalized Au NPs and fluoroquinolone antibiotics for the treatment of multidrug-resistant *Escherichia coli* infections. However, the complexity of the physicochemical properties, including size, shape, chemical modification, solvent, and environmental factors, can affect the antibacterial properties of NPs during preparation of NPs and interact with bacteria (Beyth et al., 2015). Finally, combinations of antibiotics and NPs provide complex antimicrobial mechanisms to overcome antibiotic resistance (Huh and Kwon, 2011). Gupta et al. (2017) demonstrated a synergistic effect using functionalized Au NPs and fluoroquinolone antibiotics for the treatment of multidrug-resistant *E. coli* bacterial strains.

NPs are therefore regarded as next-generation antibiotics. In both *in vitro* and *in vivo* studies, NPs, mainly metallic, have been shown to exhibit activity against gram-positive and gram-negative bacteria (Zazo et al., 2016). Though antimicrobial mechanisms that depend on the size, shape, ζ-potential, ligands, and material used are not well understood (Huh and Kwon, 2011; Singh et al., 2014; Zazo et al., 2016); currently accepted mechanisms include (1) direct interaction with the bacteria, leading to the disruption of membrane potential and integrity; (2) triggering of the host immune responses; (3) inhibition of biofilm formation; (4) generation of reactive oxygen species (ROS); and (5) inhibition of RNA and protein synthesis through the induction of intracellular effects (Pelgrift and Friedman, 2013; Beyth et al., 2015) (**Figure 1**). NP coatings on implantable devices, wound dressings, bone cement, or dental materials can function as NP-based antibiotic delivery systems (Wang et al., 2017). Furthermore, NPs can be vectors to transfer drugs so that higher doses of antimicrobial agents can be delivered to infected sites (Pelgrift and Friedman, 2013). Thus, the combination of NPs and antimicrobial agents may be beneficial in fighting the ongoing crisis of antimicrobial resistance (Baptista et al., 2018). Clinical applications of NPs have recently been evaluated to highlight the *in vitro* antimicrobial activities of NPs and the potential adverse effects of NPs on human health (**Table 1**).

**Table 1:** Nanoparticle activity against multidrug-resistant (MDR) pathogens and mechanisms of action and characteristics.

Nanoparticles (NPs)	Size	Targeted bacteria and antibiotic resistance	Antibacterial mechanisms	Factors affecting antimicrobial activity/toxicity	References
Gold (Au) NP	1–100 nm	Methicillin-resistant *Staphylococcus aureus* (MRSA)	Loss of membrane potential, disruption of the respiratory chain, reduced ATPase activity, decline in tRNA binding to ribosome subunit, bacterial membrane disruption, generation of holes in the cell wall	Roughness and particle size	(Chen et al., 2014; Dizaj et al., 2014; Rudramurthy et al., 2016; Hemeg, 2017; Zaidi et al., 2017)
Silver (Ag) NP	1–100 nm	*Staphylococcus epidermidis*, MRSA, vancomycin-resistant *Enterococcus* (VRE), extended-spectrum beta-lactamase (ESBL)-producing organisms, MDR *Escherichia coli*, *Pseudomonas aeruginosa*, *Klebsiella pneumoniae*, carbapenem- and polymyxin B-resistant *A. baumannii*, carbapenem-resistant *P. aeruginosa* and carbapenem-resistant *Enterobacteriaceae* (CRE)	Reactive oxygen species (ROS) generation, lipid peroxidation, inhibition of cytochromes in the electron transport chain, bacterial membrane disintegration, inhibition of cell wall synthesis, increase in membrane permeability, dissipation of proton gradient resulting in lysis, adhesion to cell surface causing lipid and protein damage, ribosome destabilization, intercalation between DNA bases	Particle size and shape of particles	(Dizaj et al., 2014; Cavassin et al., 2015; Rudramurthy et al., 2016; Hemeg, 2017; Zaidi et al., 2017)
Copper (Cu) NP	2–350 nm	MDR *E. coli*, *A. baumannii*	Dissipation of cell membrane potential, ROS generation, lipid peroxidation, protein oxidation, DNA degradation	Particle size and concentration	(Chatterjee et al., 2014; Dizaj et al., 2014; Cavassin et al., 2015; Hemeg, 2017; Zaidi et al., 2017)
Silica (Si) NP	20–400 nm	MRSA	Disruption of cell walls through ROS	Particle size, shape, and stability	(Dizaj et al., 2014; Zaidi et al., 2017)
Aluminum (Al) NP	10–100 nm	*E. coli*	Disruption of cell walls through ROS		(Rudramurthy et al., 2016; Hemeg, 2017)
Iron oxide NP	1–100 nm	MDR *E. coli*, *K. pneumoniae*, MRSA	ROS-generated oxidative stress: superoxide radicals (O^2−^), singlet oxygen (^1^O_2_), hydroxyl radicals (OH^−^), hydrogen peroxide (H_2_O_2_)	Has high chemical activity, tends to aggregate, is oxidized by air resulting in loss of magnetism and dispersibility	(Rudramurthy et al., 2016; Zaidi et al., 2017)
Zinc oxide (ZnO) NP	10–100 nm	*Enterobacter aerogenes*, *E. coli*, *Klebsiella oxytoca, K. pneumoniae*, MRSA, ESBL-producing *E. coli*, *K. pneumoniae*	ROS production, disruption of membrane, adsorption to cell surface, and lipid and protein damage	Particle size and concentration	(Vandebriel and De Jong, 2012; Cavassin et al., 2015; Rudramurthy et al., 2016; Hemeg, 2017)
Titanium dioxide (TiO_2_) NP	30–45 nm	*E. coli*, *P. aeruginosa*, *S. aureus*, *Enterococcus faecium*	ROS generation, adsorption to the cell surface	Crystal structure, shape, and size	(Rudramurthy et al., 2016; Hemeg, 2017)
Magnesium oxide (MgO) NP	15–100 nm	*S. aureus*, *E. coli*	ROS generation, lipid peroxidation, electrostatic interaction, alkaline effect	Particle size, pH, and concentration	(Rudramurthy et al., 2016)

## Antimicrobial Activity of NPs

NPs with antimicrobial activity that combats *Enterococcus faecium*, *Staphylococcus aureus*, *Klebsiella pneumoniae*, *Acinetobacter baumannii*, *P. aeruginosa*, and *Enterobacter* species (Ansari et al., 2014; Dizaj et al., 2014; Beyth et al., 2015; Hemeg, 2017) include NPs containing Ag, Au, Zn, Cu, Ti, Mg, Ni, Ce, Se, Al, Cd, Y, Pd, or superparamagnetic Fe (Hemeg, 2017). The antimicrobial activities against MDROs, mechanisms of action, and characteristics of various NPs are shown in **Table 1**. Among various metallic NPs and their oxides already applied as active antimicrobial agents, silver or its ionic form is the most toxic to bacteria (Seil and Webster, 2012). This makes silver of particular interest. Silver NPs (Ag NPs) are used to a great extent since they have multiple mechanisms of antibacterial action (Cheng et al., 2016), high biocompatibility, and functionalized potential and are easy to detect (Baranwal et al., 2018). Although Ag NPs are difficult to functionalize with biomolecules and antibiotics, Ag–gold (Au) alloys provide another path, since they combine the antimicrobial effects of Ag with the effectiveness of functionalization and the stability of Au in the form of bimetallic NPs (Baptista et al., 2018). Furthermore, Ag–Au NPs functionalized with tetracycline have been shown to have a synergetic effect, which is attributed to the generation of ROS (Fakhri et al., 2017).

Ag NPs and Au NPs may exhibit decreased antibacterial activity when their surfaces are modified (Rai et al., 2012; Dakal et al., 2016; Duran et al., 2016; Hemeg, 2017), and copper (Cu) NPs with modified surfaces lose antimicrobial activity and fail to change the morphology of microbial cells (Baranwal et al., 2018). However, most metallic NPs, through the release of toxic ions, inflammatory cytokines, and the generation of ROS, may cause immunotoxicity, cytotoxicity, and genotoxicity in both healthy and infected cells (Schrand et al., 2010; Ding et al., 2015).

Au–Pt bimetallic NPs have antibacterial activity against multidrug‐resistant *E. coli* through the dissipation of bacterial membrane potential and the elevation of adenosine triphosphate (ATP) levels (Baptista et al., 2018). Cu–Ni bimetallic NPs have been utilized as coating agents but have been used less in antimicrobial applications (Baptista et al., 2018)

With biocompatibility and magnetic properties, iron oxide (FeO) is well known in the biomedical sector. Recently, the antibacterial properties of reduced iron and FeO NPs that damage bacteria cells through the disruption of the bacterial membrane and generation of oxidative stress inside the cell have been studied (Baranwal et al., 2018). The characteristic compatibility and safety of ZnO NPs on human skin make them appropriate additives for cosmetics, fabrics, and surfaces in close proximity to human skin (Dizaj et al., 2014). Copper oxide (CuO) NPs have been shown to exhibit excellent bactericidal and fungicidal activity (Ren et al., 2009), whereas TiO_2_ NPs possess spectacular antimicrobial properties, mainly related to ROS formation, particularly –OH free radicals (Baranwal et al., 2018).

## Synergistic Effects of NPs with Antibiotics

To overcome antibiotic resistance, NPs can be tailored and packaged with diverse antimicrobial agents. NPs act on bacteria through multiple targets and/or a unique mechanism; thus, antimicrobial resistance is unlikely to develop if NPs are combined with antibiotics since multiple simultaneous mutations are required in the same microorganism (Fischbach, 2011; Zhao and Jiang, 2013). The functionalization of NPs with antibiotics can be a promising regimen to combat bacterial resistance. Moreover, NPs can deliver antimicrobial agents to or target the infected sites and reduce the dosage and toxicity of antibiotics (Hemeg, 2017). For example, the synergistic antibacterial efficiency of Ag NPs and antibiotics against *S. aureus*, beta-lactamase- or carbapenemase-producing *E. coli*, *P. aeruginosa*, and *A. baumannii* strains at extremely low concentrations has been found (Naqvi et al., 2013; Panacek et al., 2015; Scandorieiro et al., 2016), whereas synergistic antibacterial effects of Ag, Au, and ZnO NPs and antibiotics have been observed against *S. aureus*, *E. faecium*, *E. coli*, *A. baumannii*, and *P. aeruginosa* through the penetration of the bacterial cell membrane and the interference with important molecular pathways, formulating unique antimicrobial mechanisms (Hemeg, 2017). The efficacy of antibiotics combined with NPs was identical in both gram-positive and gram-negative bacteria, unlike the difficulty in killing MDROs with antibiotics alone (Hemeg, 2017). The combinations of antibiotics and functionalized Ag, Au, or ZnO NPs may promote the reversal of antimicrobial resistance and boost the antimicrobial effects of several antibiotics, including polymyxin B, ciprofloxacin, ceftazidime, ampicillin, clindamycin, vancomycin, or erythromycin, against MDROs, including antibiotic-resistant *A. baumannii*, *P. aeruginosa*, *E. faecium*; vancomycin-resistant *Enterococcus* (VRE); and methicillin-resistant *S. aureus* (MRSA) (Hemeg, 2017).

## Pharmacokinetic and Pharmacodynamic Characteristics of NPs

**Figure 2:**
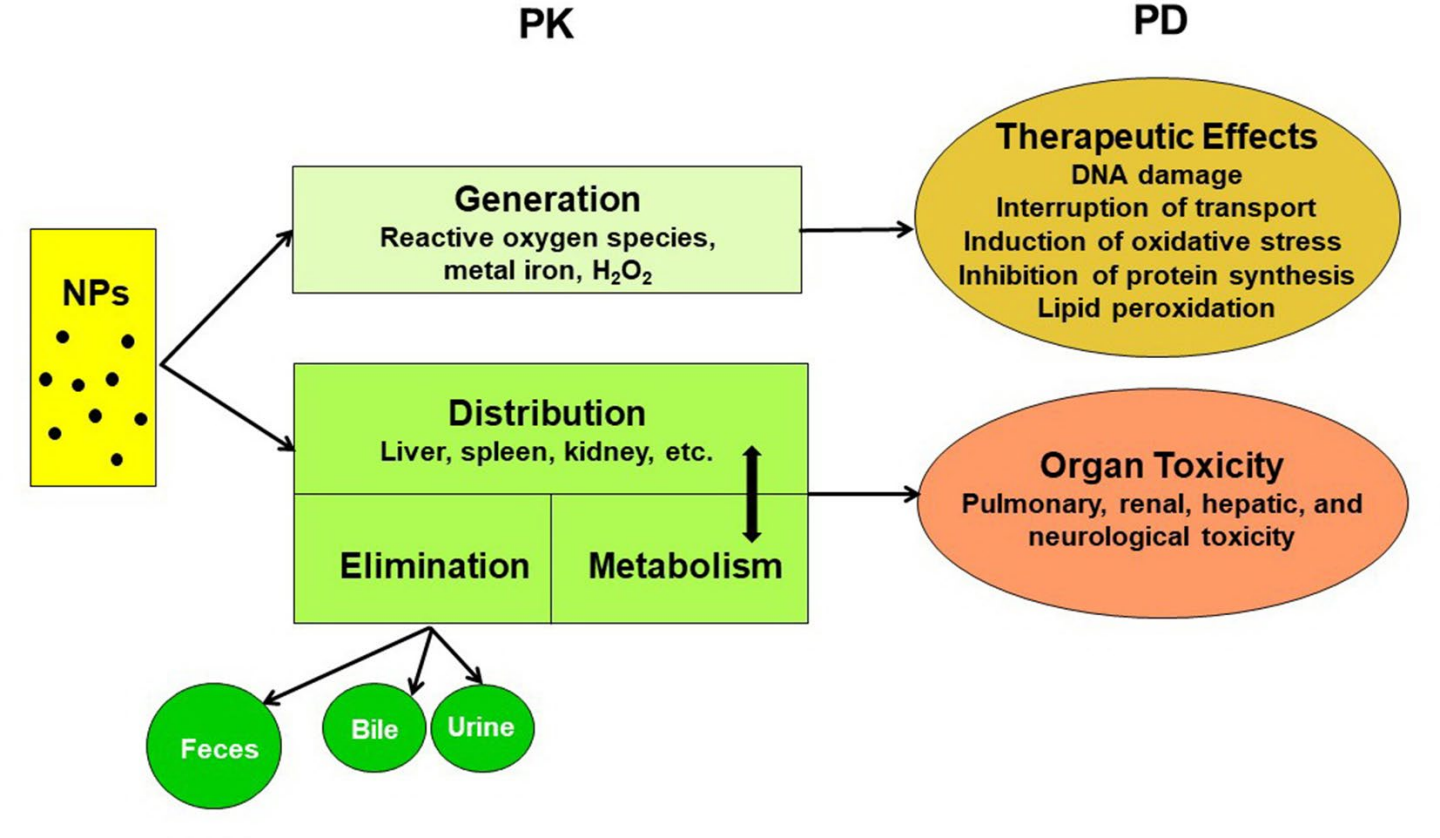
Pharmacokinetic (PK) and pharmacodynamic (PD) characteristics of nanoparticles (NPs).

The pharmacokinetics of NPs depend on numerous aspects, such as the particle type, size, surface charge, surface coating, protein binding, exposure route, dose, and animal species. A comprehensive understanding of their pharmacokinetics is pivotal for risk assessment and biosafety in clinical practice (Lin et al., 2015). The pharmacokinetic and pharmacodynamic characteristics of NPs are summarized in **Figure 2**. The systemic or local activity and toxicity of NPs are dependent on the administration route and physicochemical characteristics, and chronic toxicity may be related to the complicated elimination pathway (Zazo et al., 2016). A summary of the present knowledge of the pharmacokinetics and toxicity of metallic NPs is provided in **Table 2**.

**Table 2:** Comparisons of the pharmacokinetic characteristics and toxicity of metallic nanoparticles (NPs).

Type of NP	Absorption	Tissue distribution	Metabolism	Elimination	Toxicity
Au NP	*T_1/2_: increases with decreasing particle size	*Distributed to the liver (51.3–96.9%) and spleen (2–11.4%) after venous injection	Degraded within the endosomal compartments in mammalian cells	*Low renal elimination: 9% for 1.4-nm Au NPs within 24 h after parenteral injection (rats)	Cellular membrane toxicity
	*Low oral absorption: 0.37–0.01% for large size	*Crosses the blood–brain barrier (BBB) to a low extent			
	*Negatively charged Au NPs have a higher absorption than positive particles (0.37% *vs.* 0.14%, respectively; 2.8 nm) in rats	*Placental transfer depends on both the stage of embryonic/placental maturation and the surface composition		*Renal elimination: more efficient than biliary excretion, if size < the threshold value of 5.5 nm	
	*Inhalational absorption: 0.06–5.5%	*Distributes to tissues and remains for a long time (>6 months)		*Biliary excretion is higher than urinary excretion if size = 13 nm	
Ag NP	T_1/2_: 4.1 days (rats) and 11.7–16.3 days (rabbits) for 7.9 nm after parenteral injection	*Mainly distributed to liver and spleen followed by kidneys, regardless of the exposure route	*Release Ag^+^, which can precipitate with Cl^−^ in the stomach	Elimination profiles: size <5.5 nm not reported; >5.5 nm: biliary elimination is more efficient than urine elimination	Allergies; cytotoxicity; neurologic, renal, hepatic, and blood cell complications; skin discoloration; mitochondrial toxicity; and oxidative stress in brain tissue
		*≥80 nm: mainly distributed to the spleen; ≤60 nm mainly accumulated in the liver			
		*Both Ag NPs and Ag ions pass the BBB, but micronized Ag particles cannot: Ag NPs have been detected in neuronal cells	*In blood, Ag^+^ can bind to proteins with thiol groups and distribute to various tissues		
Other metallic NPs	*T_1/2_ of FeO NPs: shorter than Au NPs and Ag NPs (ferumoxtran-10: 2.6 h in rats and 5.7 h in monkeys)	The liver and spleen are also the primary organs of distribution for FeO NPs, TiO_2_ NPs, and ZnO NPs	FeO NPs: progressively degraded and eliminated primarily in urine; >80% remain in the body for 84 days after exposure	*FeO NPs: elimination is mainly *via* the biliary route, excreted in urine and feces	*High toxicity of CuO NPs causes oxidative lesions
					*DNA damage induced by ZnO and TiO_2_ NPs
	*Low oral, dermal, or pulmonary absorption of TiO_2_ NPs	TiO_2_ NPs are able to cross the placenta to reach the fetus and stay in the offspring’s brain up until at least the juvenile period		*TiO_2_ NPs: more excretion *via* urine than *via* feces	*ZnO NPs cause lung inflammation and systemic toxicity
Remarked issue	Limited pharmacokinetic data of metallic NPs in large laboratory animals (e.g., pigs)	Limited information about the effects of physicochemical properties of metallic NPs on specific tissue distribution	*No published *in vivo* studies on the metabolism of Au NPs	Limited data to increase biliary and/or urine excretion of metallic NPs	No conclusive evaluation of the exact mechanism for NP toxic complications
			*Enzymes that mediate metabolism or degradation of metallic NPs have yet to be identified		

*The asterisks indicate the different subitems, and there will be no asterisk if there was one subitem.

The oral, dermal, or pulmonary absorption of Au NPs, Ag NPs, or TiO_2_ NPs is generally low (**Table 2**). The parenteral route is favored for targeting the liver or spleen. For muscle or skin targeting, the administrative route can be topical, intramuscular, intradermal, or subcutaneous, whereas oral or intranasal administration is used in the case of mucosal targeting (Zazo et al., 2016). For example, the absorption of Au NPs by inhalation ranges from 0.06% to 5.5%, depending on the size of the NP (Lin et al., 2015; Zazo et al., 2016). Oral absorption is approximately 0.01–5% for Au NPs, 1–4.2% for Ag NPs, and 0.01–0.05% for TiO_2_ NPs, depending on the size and coating (Lin et al., 2015).

Regardless of the particle type, most metallic NPs are distributed mainly in the liver and spleen, but the physicochemical properties of Au NPs could be modified to increase their distribution to specific target organs (Lin et al., 2015). However, long-term studies regarding a full interpretation of the toxicological implications of NP absorption and penetration through tissues are lacking (Lin et al., 2015).

### Dose Optimization

The decision for the optimal dose is crucial for therapeutic targets and minimizing toxicity for medical translation (Khan et al., 2016; Hua et al., 2018). Thus far, the doses of nanomaterials causing cell damage *in vitro* are unrealistically high and are impossible to apply to humans (Khan et al., 2016). The data from animal studies may not be directly translated to human beings, and appropriate and realistic doses should be studied in the future (Khan et al., 2016; Hua et al., 2018). There have been few clinical studies on NP dosing. Munger et al. (2014) reported two oral doses (10 ppm with a size ranging from 5 to 10 nm and 32 ppm with a size ranging from 25 to 40 nm) of a commercial solution of Ag NP in healthy adult volunteers that did not prompt clinically significant changes in human metabolic and hematologic profiles, urine, physical findings, or imaging morphology based on comprehensive assays and tests. More clinical studies are warranted before the application of NPs to patients.

### Clearance and Elimination

The elimination of metallic NPs *via* urinary and biliary pathways is generally low, which leads to their long-term accumulation in the liver and spleen (Lin et al., 2015). In addition, NPs do not undergo biodegradation into biologically benign components and thus exhibit prolonged tissue retention, eventually leading to amplified toxic effects (Zaidi et al., 2017). A higher accumulation of 10-nm NPs was observed in the kidneys, but this could be caused by a lessened availability of the larger NPs due to their high accumulations in the liver and spleen (Hoshyar et al., 2016).

The degree of opsonization of NPs by serum proteins is determined by the charge and size of the NPs. By opsonization, the *in vivo* hydrodynamic diameter (HD) or the effective size of NPs can be altered (Zaidi et al., 2017). The endothelium usually has a pore size of 5 nm, and particles with an HD smaller than 5 nm can equilibrate with the extravascular extracellular space (EES). Conversely, larger particles with slow movement across the endothelium remain in circulation for extended periods (Zaidi et al., 2017). The kidney can remove molecules from vascular compartments, but the particles in the range of 10–20 nm are excluded from renal filtration and are eliminated through the hepatobiliary system (Zaidi et al., 2017). The remaining particles that escape degradation by Kupffer cells will be retained in the body for prolonged periods (Zaidi et al., 2017). More studies are vital to explore the ways to increase biliary and/or urine elimination of NPs to reduce organ accumulation and potential toxicity (Lin et al., 2015).

### Pharmacodynamic Properties

The antimicrobial activity of NPs depends on several physicochemical properties, such as their size, shape, solubility, and ability to form free biocidal metal ions (Khan et al., 2016). Generally, smaller NPs show increased antibacterial activity compared to larger NPs (Lu et al., 2013). Gram-positive and gram-negative bacteria differ in terms of cell membrane components and structures and have different adsorption pathways for NPs (Lesniak et al., 2013). The susceptibility of bacteria to NPs depends on their biochemical composition since different NPs target different biomolecules (Khan et al., 2016). Moreover, rapidly growing bacteria are more susceptible to NPs or antibiotics than slow-growing bacteria. This may be due to the variable expression of stress-response genes between rapidly growing and slow-growing bacteria (Stewart, 2002; Khan et al., 2016).

The antibacterial effects of NPs have been noted to be more pronounced for gram-positive bacteria than for gram-negative bacteria. Such a finding may be related to the fact that the nonporous cell walls of gram-negative bacteria serve as penetration barriers for the entry of NPs (Zaidi et al., 2017). Cell walls of gram-positive bacteria with covalent links with neighboring proteins and components are relatively porous and allow the penetration of foreign molecules (Zaidi et al., 2017).

## Toxicity

Local and systemic toxic complications, as well as deleterious effects on beneficial bacteria in humans, are concerns for the use of NPs (Zhang et al., 2010; Khan et al., 2016). Both NPs themselves and toxic degradation products of NPs can cause hemolysis and interfere with blood coagulation pathways (Kandi and Kandi, 2015). The exact mechanism of toxic complications is unclear, but it has been observed that the larger the size of the NP is, the greater the risk of adverse health effects (Dos Santos et al., 2014). Among metal NPs, the toxicity of Ag NPs has been studied extensively, and Ag NPs were shown to be more toxic toward cell lines. However, most studies were performed *in vitro* (Bondarenko et al., 2013; Ivask et al., 2014). The deposition of Ag NPs in the liver, spleen, lungs, and other organs results in organ damage and dysfunction and seriously decreases their efficacy (Hemeg, 2017). Elevated Ag levels have been found in both blood and urine by the leaching of Ag from Acticoat^®^, a nanocrystalline Ag wound dressing, into the bloodstream (Khan et al., 2016) and were confirmed in burn patients (Vlachou et al., 2007). Al_2_O_3_ NPs that interact with cellular biomolecules and cause adverse effects of neurotoxicity could serve as broad-spectrum bactericidal agents, regardless of drug resistance mechanisms (Ansari et al., 2014). The oxidative damage of CuO NPs and DNA damage induced by ZnO NPs or TiO_2_ NPs limit their use (Hemeg, 2017).

Intravenously administered NPs could accumulate in the colon, lung, bone marrow, liver, spleen, and lymphatic system (Hagens et al., 2007), and inhalation might cause cytotoxicity in the lung (Leucuta, 2013). The generated free radical-mediated oxidative stress by CuO NP could interact with cell components and induce hepatotoxicity and nephrotoxicity (De Jong and Borm, 2008; Lei et al., 2008; Baptista et al., 2018). Though several *in vivo* studies have reported no apparent life-threatening toxicity related to NPs (Pfurtscheller et al., 2014; Sengupta et al., 2014; Wei et al., 2015; Zazo et al., 2016), chronic toxicity, such as nephrotoxicity, hepatotoxicity, or pulmonary toxicity, can result from the accumulation of metallic NPs in these tissues (Duncan and Gaspar, 2011; Arvizo et al., 2012; Wei et al., 2015; Zazo et al., 2016).

However, the evaluation of toxicity at the cellular and systemic levels remains important for clinical translation, and several parameters, such as the administration route for a desired therapeutic effect (Khan et al., 2016) and the nature and extent of the interactions between NPs and cells, tissues, and organs, should be considered (Sandhiya et al., 2009). Detailed *in vivo* and clinical studies assessing the toxicity of NPs are highly desirable before the routine application of NPs in combating difficult-to-treat infections due to MDROs.

## Resistance to NPs

NPs have multifunctional mechanisms to attack bacteria that are different from those of the currently available antibiotics (**Figure 1**), and the combination of NPs and clinically available antibiotics allows for recovery of antimicrobial efficacy (Zhao and Jiang, 2013; Zazo et al., 2016). Microbial cells need to acquire multiple mutations to develop resistance toward NPs (Singh et al., 2018). Furthermore, the synthesis of NPs that bind with proteins, polysaccharides, or small bioactive compounds would further enhance their antimicrobial activity toward MDROs (Singh et al., 2018). Resistance to NPs is always a clinical concern (Zhao and Jiang, 2013). Though rare, bacteria resistant to Ag, Au, or Cu NPs have been reported even after exposure to one dose of NPs (Zhao and Jiang, 2013; Finley et al., 2015; Zazo et al., 2016). The resistance might be related to changes in the permeability of the outer membrane and high expression of efflux pumps (Zhao and Jiang, 2013; Finley et al., 2015).

Another example of resistance to NPs is that after exposure to Cu^++^ and Cu-doped TiO_2_ NPs, reduced antimicrobial activity of TiO_2_ NPs to *Shewanella oneidensis* was noted. This effect is likely to be associated with decreased uptake and/or increased efflux of Cu^++^ and Cu-doped TiO_2_ NPs (Wu et al., 2010; Hajipour et al., 2012). Reduced toxic effects of both TiO_2_ and Al_2_O_3_ NPs to *Cupriavidus metallidurans* were possibly due to less uptake of plasma membrane or cell wall or increased efflux of NPs (Pelgrift and Friedman, 2013).

The increasing clinical application of Ag NPs still raises the concern of bacterial resistance to Ag NPs (Barros et al., 2018). Resistance to Ag NPs attributed to *sil* genes has been reported in clinical *K. pneumoniae* and *Enterobacter cloacae* isolates from burn cases (Finley et al., 2015). Genetic changes in bacteria may result in the rapid evolution of resistance to Ag NPs (Graves et al., 2015), and Al_2_O_3_ NPs could trigger increased expression of conjugation-promoting genes and promote the horizontal transfer of antibiotic resistance genes (Hemeg, 2017). The phenotypic change in the production of flagellin in *E. coli* isolates resistant to Ag NPs was found to readily induce NP aggregation and attenuate the antimicrobial activity of Ag NPs (Finley et al., 2015; Panacek et al., 2018).

## Strengths and Limitations of the Application of NPs Against MDROs

NPs have the potential to treat bacterial infections (**Table 3**), but several challenges remain for their successful translation to the clinic, including further assessment of the interactions of NPs with cells, tissues, and organs; optimal dose; recognition of appropriate administration routes; and toxicity following acute and long-term exposure (Sandhiya et al., 2009; Huh and Kwon, 2011; Baptista et al., 2018).

**Table 3:** Advantages and disadvantages of antimicrobial nanoparticles.

Advantages	Disadvantages
Targeted drug delivery *via* specific accumulation	Accumulation of intravenously injected nanomaterials in tissues and organs
Fewer side effects of chemical antimicrobials	High systemic exposure to locally administered drugs with proper doses for desirable therapeutic use
Less prone to bacterial resistance	High systemic exposure to locally administered drugs with proper doses for desirable therapeutic use
Can cross tissue barriers (e.g., blood–brain barrier)	
Extended therapeutic lifetime due to slow elimination	Nanotoxicity (lung, kidney, liver, brain, germ cell, metabolic, etc.)
Controlled drug release	
Broad therapeutic index	Lack of characterization techniques that are not affected by the properties of nanoparticles (NPs)
Improved solubility	
Low immunosuppression	

The unique physical structure of NPs offers distinctive advantages over conventional antibiotics in terms of antibiotic resistance (Zazo et al., 2016). The current state of NPs exhibits a strong potential to topically treat skin infections in the near future (Zazo et al., 2016). Efforts have been made to apply NPs on the contact surfaces of medical devices, fibers, and textiles (Zazo et al., 2016). However, systemic administration of NPs still requires multiple aspects to be addressed (Zazo et al., 2016; Zaidi et al., 2017).

Formulation of proper guidelines for the production and scaled-up manufacturing of these nanomaterials, the characterization of the physicochemical properties and their effect on biocompatibility, standardization of nanotoxicological assays, and protocols to compare data originating from *in vitro* and* in vivo* studies are urgent for clinical translation (Duncan and Gaspar, 2011; Beyth et al., 2015; Rai et al., 2016; Zazo et al., 2016). Further preclinical studies have to consider the therapeutic efficacy parameters in clinical trials and the safety of NP systems (Zazo et al., 2016). Finally, the economic impact of clinical translation of these NPs must be addressed with regard to their therapeutic efficacy (Duncan and Gaspar, 2011; Zazo et al., 2016).

## Conclusion and Future Directions

Given their therapeutic potential, it is essential to determine the mechanisms by which NP complexes inhibit or kill bacteria. However, there is limited information about the metabolism, clearance, and toxicity of NPs; the nature of optimal targets for certain infections; and the optimum dose for therapeutic activity at the pathogen target sites. Specific combinations of NPs and antibiotics can prevent the emergence of resistance or drive resistant bacteria back toward drug sensitivity, but translation into the clinic requires an in-depth perception of the pharmacokinetics/pharmacodynamics of NPs.

## Author Contributions

NL wrote the manuscript, and WK and PH revised and approved the final version of the manuscript.

## Funding

This study was supported by the grants from National Cheng Kung University Hospital, Tainan, Taiwan (NCKUH-10802042) for publication fee.

## Conflict of Interest

The authors declare that the research was conducted in the absence of any commercial or financial relationships that could be construed as a potential conflict of interest.
